# Resting state brain networks arise from electrophysiology-invisible signals

**DOI:** 10.21203/rs.3.rs-3251741/v1

**Published:** 2023-08-14

**Authors:** Nanyin Zhang, Wenyu Tu, Samuel Cramer

**Affiliations:** Pennsylvania State University; Pennsylvania State University; Pennsylvania State University

**Keywords:** resting-state fMRI, electrophysiology, rat

## Abstract

Resting-state brain networks (RSNs) have been widely applied in health and disease, but their interpretation in terms of the underlying neural activity is unclear. To systematically investigate this cornerstone issue, here we simultaneously recorded whole-brain resting-state functional magnetic resonance imaging (rsfMRI) and electrophysiology signals in two separate brain regions in rats. Our data show that for both recording sites, band-specific local field potential (LFP) power-derived spatial maps can explain up to 90% of spatial variance of RSNs obtained by the blood-oxygen-level dependent (BOLD) signal. Paradoxically, the time series of LFP band power can only explain up to 35% of temporal variance of the local BOLD time course from the same location even after controlling for the factors that may affect apparent LFP-BOLD correlations such as contrast-to-noise ratio. In addition, regressing out LFP band powers from the rsfMRI signal does not affect the spatial patterns of BOLD-derived RSNs, collectively suggesting that the electrophysiological activity has a marginal effect on the rsfMRI signal. These findings remain consistent in both light sedation and awake conditions. To reconcile this contradiction in the spatial and temporal relationships between resting-state electrophysiology and rsfMRI signals, we propose a model hypothesizing that the rsfMRI signal is driven by electrophysiology-invisible neural activities that are active in neurovascular coupling, but temporally weakly correlated to electrophysiology data. Meanwhile, signaling of electrophysiology and electrophysiology-invisible/BOLD activities are both constrained by the same anatomical backbone, leading to spatially similar RSNs. These data and the model provide a new perspective of our interpretation of RSNs.

## Introduction

Sophisticated brain function requires coordinated activities from separate brain regions, which collectively constitute functional brain networks. Functional brain networks in humans and animals are predominantly studied using the method of resting-state functional magnetic resonance imaging (rsfMRI), which quantifies the synchronization of brain-wide spontaneous blood-oxygen-level dependent (BOLD) signals, and such networks are termed as resting-state brain networks (RSNs).

Although BOLD-derived RSNs have been widely applied in health and disease, their relationship to the underlying neural activity is not fully understood. This is a fundamental issue, because the BOLD signal is known to not directly, but indirectly reflect neural activity via the accompanied hemodynamic/metabolic changes, a mechanism called neurovascular coupling (NVC). Although tight NVC has been repeatedly demonstrated when neural activities are evoked by explicit external stimulation (Goense and Logothetis, 2008; Logothetis et al., 2001; Nemoto et al., 2004), this relationship in the resting state remains elusive. On the one hand, substantial evidence demonstrates that the spatial patterns of most RSNs well replicate known functional systems and activation patterns elicited by various tasks (Biswal et al., 1995; Glasser et al., 2016; Hampson et al., 2002; Lowe et al., 1998; Smith et al., 2009), as well as patterns of structural networks (Andrews-Hanna et al., 2010; Greicius et al., 2009; Lowe et al., 2008). In addition, alterations in RSNs are reported in various brain disorders that correspond to neuropathophysiological changes (Buckner et al., 2009), collectively suggesting that RSNs must have strong neural basis. On the other hand, the time series of resting-state electrophysiological signal often has fairly low prediction power of the corresponding rsfMRI time series (Mateo et al., 2017; Scholvinck et al., 2010; Winder et al., 2017), and some studies even show a disconnect between neural activity and hemodynamic signals in certain conditions (Maier et al., 2008; Zhang et al., 2019). In addition, different studies reported mixed electrophysiology correlates of the rsfMRI signal across LFP bands in a wide frequency range, from infraslow signal (Hiltunen et al., 2014; Pan et al., 2013), low-frequency delta/sub-delta band signal (He et al., 2008; Lu et al., 2007) to high-frequency gamma band signal (Bastos et al., 2015; Foster et al., 2015; Keller et al., 2013; Mukamel et al., 2005; Nir et al., 2008) and spiking activity (Mukamel et al., 2005), which indicate that the rsfMRI signal may reflect different aspects of neural activity such as synaptic current and spiking. Taken together, it is far from being clear how RSNs and rsfMRI are related to spontaneous neural activity (Laufs et al., 2003; Liu et al., 2011; Mantini et al., 2007), and lack of such knowledge represents a major knowledge gap in our ability to interpret functional brain networks.

To address this critical issue, we systematically examined the role of electrophysiology activity in determining specific spatiotemporal patterns of BOLD-based RSNs. Along with whole-brain rsfMRI, electrophysiology signals were simultaneously recorded in the primary motor cortex (M1) and anterior cingulate cortex (ACC) in rats under both light sedation and awake conditions. M1 and ACC were selected given their distinct role in sensorimotor function and integrative cognition, respectively. Our data show that in both light-sedation and awake states, the gamma band power-derived RSN spatial patterns highly resemble BOLD-derived RSNs for both recording sites, while lower frequency band-derived RSNs exhibit inversed spatial patterns, both indicating strong neural underpinning of RSNs. Paradoxically, at both recording sites, band-limited LFP powers display considerably lower temporal correlations with the corresponding local BOLD signals. Further, regressing out the gamma band power or powers of all LFP bands did not change BOLD-derived RSNs, suggesting LFP powers only play a marginal role in the rsfMRI signal. These paradoxical findings imply that there must be electrophysiology-invisible brain activities that have major contributions to the rsfMRI signal and RSNs.

## Results

**Figure 1:**
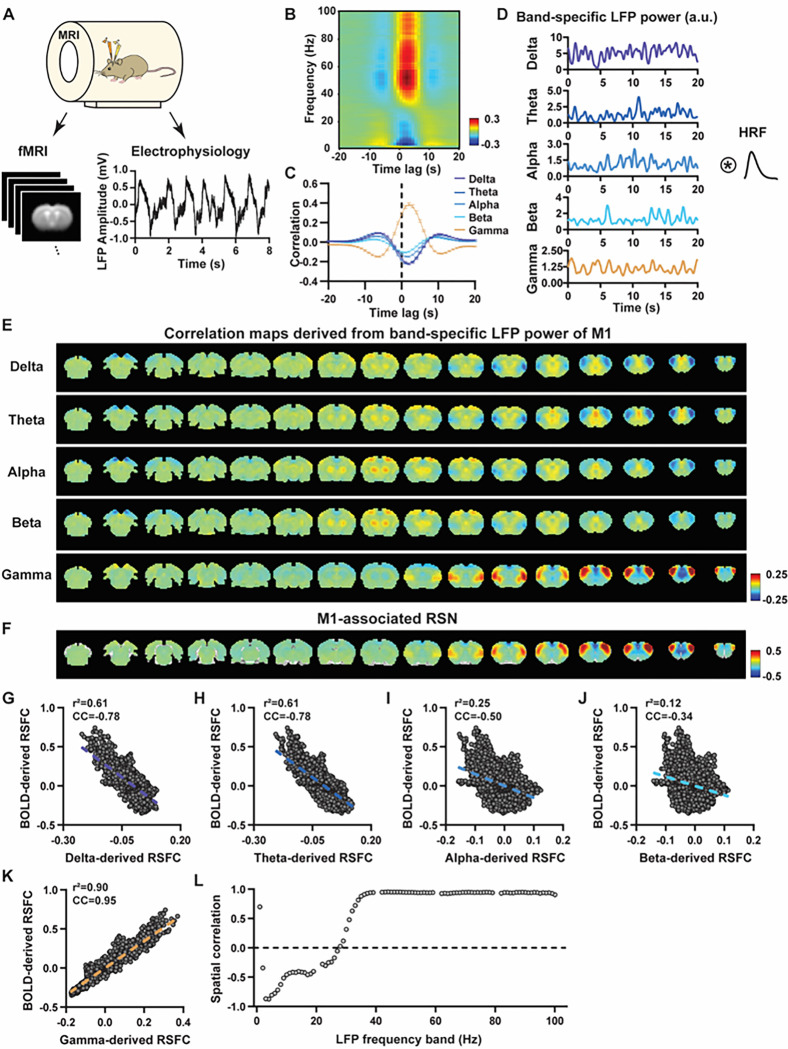
LFP and rsfMRI signals in the M1 derive highly consistent RSN spatial patterns in lightly sedated rats. **A)** Simultaneous acquisition of whole-brain rsfMRI and electrophysiological signals in the M1 and ACC. **B)** Cross correlations between the rsfMRI signal and LFP power in the M1 (0.1–100 Hz, band interval: 1Hz, lag range: −20 – 20 s. **C)** Cross correlations between the rsfMRI signal and powers of individual LFP bands in the M1. Bars: SEM**. D)** Exemplar powers of individual LFP bands. Convolving powers of individual LFP bands with a rodent-specific hemodynamic response function (HRF) generates the corresponding LFP-predicted BOLD signals. **E)** Correlation maps derived by band-specific LFP powers in the M1, obtained by voxel-wise correlating the LFP-predicted BOLD signal for each band with BOLD signals of all brain voxels. **F)** M1 seedmap, obtained by voxel-wise correlating the regionally averaged BOLD time course of the seed (i.e. M1) with BOLD time courses of all brain voxels**. G-K)** Spatial similarity between the M1 seedmap and the M1 LFP-derived correlation map of each band, quantified by their voxel-to-voxel correlations (G: delta, CC = −0.78; H: theta, CC = −0.78; I: alpha, CC = −0.5; J: beta, CC = −0.34; K: gamma, CC = 0.95). **L)** Spatial correlations between the M1 seedmap and correlation maps derived by individual 1-Hz bands in the full LFP spectrum.

To systematically analyze the spatiotemporal relationship between resting-state electrophysiology and fMRI signals, we simultaneously recorded the whole-brain rsfMRI and electrophysiology signals in the M1 and ACC in rats in both light-sedation and awake states (Fig. 1A). The placement of implanted electrodes in the M1 and ACC was verified using T2-weighted structural images (Fig. S1). Raw electrophysiology data were initially preprocessed to remove MR artifacts using a template regression approach (Tu and Zhang, 2022). An example of denoised LFP is shown in Fig. 1A and Fig. S2.

### LFP and rsfMRI signals derive highly consistent RSN spatial patterns in lightly sedated rats

**Figure 2:**
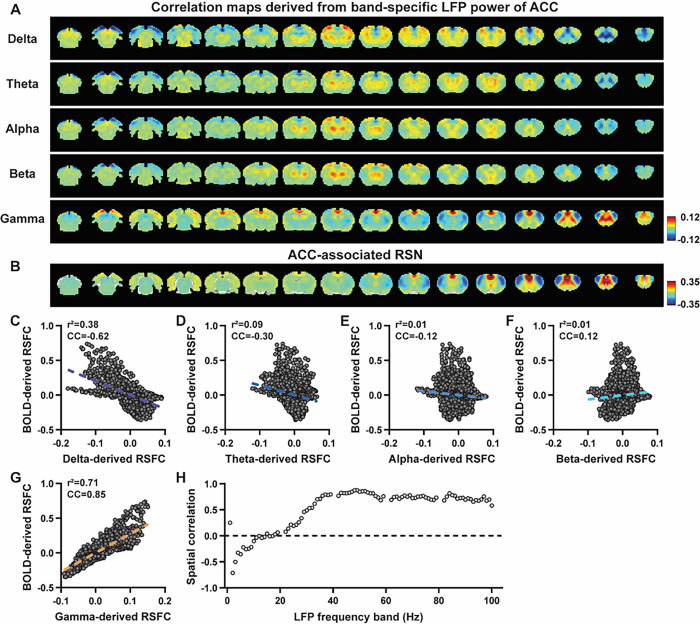
LFP and rsfMRI signals in the ACC derive highly consistent RSN spatial patterns in lightly sedated rats. **A)** Correlation maps derived by band-specific LFP powers in the ACC, obtained by voxel-wise correlating the LFP-predicted BOLD signal for each band with the BOLD signals of all brain voxels. **B)** ACC seedmap, obtained by voxel-wise correlating the regionally averaged BOLD time course of the seed (i.e. ACC) with BOLD time courses of all brain voxels. **C-G**) Spatial similarity between the ACC seedmap and the ACC LFP-derived correlation map of each band, quantified by their voxel-to-voxel correlations (C: delta, CC = −0.62; D: theta, CC = −0.30; E: alpha, CC = −0.12; F: beta, CC = 0.12; G: gamma, CC = 0.85). **H**) Spatial correlations between the ACC seedmap and correlation maps derived by individual 1-Hz bands in the full LFP spectrum.

We first examined the spatial relationship between brain-wide rsfMRI signals and frequency band-specific LFP powers in lightly sedated rats. For each recording site, its associated RSN is obtained as the seedmap, calculated by voxel-wise correlating the regionally averaged BOLD time series of the seed (M1 in Fig. 1F and ACC in Fig. 2B) with BOLD time series of individual brain voxels. This map is conventionally defined as the resting-state functional connectivity (RSFC) pattern for the seed. To determine to what extent this RSN can also be derived using the LFP signal recorded from the same location (M1 or ACC), the band-limited LFP power was generated using a conventional LFP band definition (delta: 1–4 Hz, theta: 4–7 Hz, alpha: 7–13 Hz, beta: 13–30 Hz, gamma: 40–100 Hz). Figures 1B&C show cross correlations of the LFP power and BOLD signal in the M1 across the LFP spectrum (Fig. 1B, 1-Hz band interval) and for individual LFP bands (Fig. 1C), indicating that gamma band power is positively correlated with the BOLD signal, while lower-frequency bands display negative peak correlations with the BOLD signal. The lag of the BOLD signal is approximately 2 sec for all bands, consistent with the hemodynamic response function (HRF) previously reported in rodents (Scholvinck et al., 2010; Winder et al., 2017). Therefore, for each LFP band, we convolved its power with an rodent-specific HRF (Fig. 1D, (Tong et al., 2019)) to generate the LFP band-predicted BOLD signal, which was then voxel-wise correlated with the brain-wide rsfMRI signal, yielding the LFP band-derived correlation map (Fig. 1E, Fig. 2A).

We found that the gamma band power-derived correlation map is spatially highly consistent with the corresponding seedmap. For M1, the voxel-to-voxel Pearson correlation coefficient (CC) between the mean gamma-derived correlation map (Fig. 1E) and mean M1 seedmap (Fig. 1F) is 0.95 (Fig. 1K, R^2^ = 0.90), indicating 90% of the variance in the M1 seedmap can be explained by the gamma-derived map. In contrast, the spatial maps generated by lower-frequency bands are inversely correlated with the M1 seedmap, with a trend that CC being more negative as the frequency range of the band is lower (Figs. 1E–J, delta: CC = −0.78; theta: CC = −0.78; alpha: CC = −0.5; beta: CC = −0.34), consistent with the LFP-BOLD cross correlations for these bands shown in Fig. 1B–C. These relationships are repeatable in the ACC (Figs. 2A–G, delta: CC = −0.62; theta: CC = −0.3; alpha: CC = −0.12; beta: CC = 0.12; gamma: CC = 0.85). These data indicate that the spatial patterns of BOLD-based RSNs can be reliably obtained using the band-specific LFP signal.

To examine whether these findings happen to result from the specific frequency cutoffs we adopted for any LFP band, we repeated the analysis for all individual 1-Hz bands in the full LFP spectrum. Our data again show a gradual transition from negative to positive spatial correlations in LFP-derived correlation maps with the corresponding seedmap as the LFP signal increases from low to high frequencies (Fig. 1L for M1; Fig. 2H for ACC). Furthermore, as a control analysis, the gamma band power in the ACC was temporally shuffled, convolved with the HRF and the correlation map was recalculated (Fig. S3). In this case the spatial pattern observed in Fig. 2A disappeared, suggesting the LFP-derived spatial patterns are indeed specifically related to the LFP signal, instead of an artifact of HRF. We also confirmed that all our results are not sensitive to the rsfMRI data preprocessing step of global signal regression (Figs. S4-S5).

These data collectively indicate the same RSNs can be reliably derived using LFP power in lightly sedated rats, suggesting the critical involvement of neural activity in RSN spatial patterns.

### Temporal correlations between LFP power and local rsfMRI signal are significant but considerably weaker

**Figure 3:**
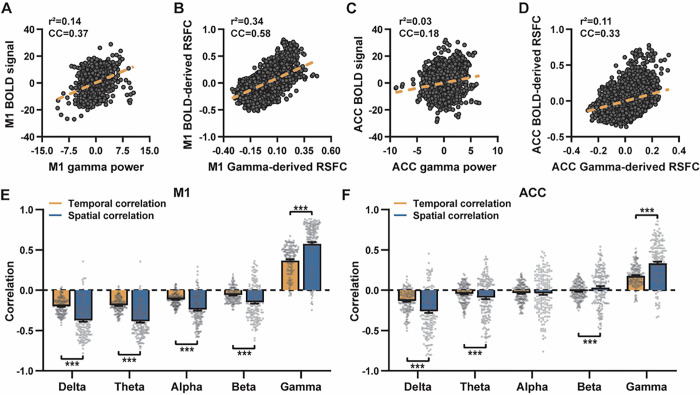
Disparity in spatial and temporal correlations persists after controlling for signal variance. **A, C)** Representative volume-to-volume temporal correlations between the HRF-convolved gamma powers and local rsfMRI signals from two individual scans. These scans are selected because they have the same temporal correlation values as the group averages for the **A)** M1 (R = 0.37) and **C)** ACC (R = 0.18), respectively. **B, D)** Representative voxel-to-voxel spatial correlations between gamma-derived correlation maps and seedmaps from two scans with the same spatial correlation values as the group averages for **B)** M1 (R = 0.58) and **D)** ACC (R = 0.33). **E, F)** Comparison of scan-wise spatial and temporal correlations (paired t tests across individual scans; **E)** M1 (# of scans = 159), delta: p = 1.01 × 10^−35^; theta: p = 3.74 × 10^−50^; alpha: p = 3.54 × 10^−25^; beta: p = 1.18 × 10^−12^; gamma: p= 7.02 × 10^−42^; F) ACC (# of scans = 172), delta: p = 6.73 × 10^−21^; theta: p = 5.75 × 10^−5^; alpha: p = 0.74; beta: p = 7.34× 10^−5^; gamma: p=3.43 × 10^−29^ ).***:p<0.005.

Given the high reliability of the gamma power in determining the spatial patterns of BOLD-based RSNs, it is tempting to assume the HRF-convolved gamma power ought to be able to reliably predict the rsfMRI time series from the same location (Fig. 3A&C). Surprisingly, our data show that the local rsfMRI signal displays considerably weaker temporal correlations with LFP powers. In the M1, the LFP-BOLD temporal correlations gradually change from negative to positive as the LFP signal goes from low to high frequencies—the same trend observed in spatial correlations (Figs. 1E–K), with, however, significant but considerably lower absolute magnitude in CC values (one sample t-tests, delta: CC = −0.20, p = 2.1 × 10^−57^; theta: CC = −0.19, p = 7.1 × 10^−62^; alpha: CC = −0.11, p = 1.2 × 10^−42^; beta: CC = −0.06, p = 8.4 × 10^−15^; gamma: CC = 0.37; p = 2.1 × 10^−58^; number of scans = 159). The same results are observed in the ACC (one sample t-tests; delta: CC = −0.13, p = 1.9 × 10^−28^; theta: CC = −0.04, p = 8.4 × 10^−7^; alpha: CC = −0.03, p = 6.0 × 10^−5^; beta: CC = −0.01, p = 0.1; gamma: CC = 0.18, p = 6.7 × 10^−42^; number of scans = 172).

Notably, one difference in our calculation of spatial and temporal correlations may contribute to the disparity in their CC values. In our calculation of spatial correlations, we first scan-wise generated LFP- and BOLD-derived RSN maps, and these maps were then averaged within each group before the spatial correlations were calculated using two averaged maps (Figs. 1–2). However, the temporal correlations were first calculated for individual scans (Figs. 3A&C) before the resulting correlation values were averaged across scans, as averaging time courses first would diminish the actual signal due to the semi-random nature of spontaneous brain activities. As a result, the variance in averaged RSN spatial maps might be lower than the variance in time series of individual scans, which can result in higher apparent spatial correlations than temporal correlations. To control for this factor, we also calculated spatial correlations between LFP- and BOLD-derived maps for individual scans (Figs. 3B&D), and then averaged the corresponding correlation values across scans. Like scan-wise temporal correlations, scan-wise spatial correlations are significant for all LFP bands (one sample t-tests; in the M1, delta: CC = −0.37, p = 4.9 × 10^−52^; theta: CC = −0.39, p = 3.9 × 10^−62^; alpha: CC = −0.24, p = 5.8 × 10^−37^; beta: CC = −0.15, p = 3.9 × 10^−15^; gamma: CC = 0.58, p = 1.0 × 10^−56^; in the ACC, delta: CC = −0.26, p = 2.6 × 10^−27^; theta: CC = −0.09, p = 1.7 × 10^−6^; alpha: CC = −0.04, p = 0.04; beta: CC = 0.03, p = 0.06; gamma: CC = 0.33; p = 5.4 × 10^−40^). Comparisons of scan-wise spatial and temporal correlations are shown in Figs. 3E&F (paired t tests across individual scans; in the M1, delta: p = 1.01 × 10^−35^; theta: p = 3.74 × 10^−50^; alpha: p = 3.54 × 10^−25^; beta: p = 1.18 × 10^−12^; gamma: p = 7.02 × 10^−42^; in the ACC, delta: p = 6.73 × 10^−21^; theta: p = 5.75 × 10^−5^; alpha: p = 0.74; beta: p = 7.34× 10^−5^; gamma: p = 3.43 × 10^−29^). These data show that even after controlling for the variance level, spatial correlations are still appreciably higher than temporal correlations.

It is likely that true LFP-BOLD temporal correlations are actually high, and lower apparent temporal correlations we observed are simply because the rsfMRI signal is noisy. Even though we cannot average rsfMRI/electrophysiology time courses across scans to reduce the noise level, we ask whether we can still estimate the true LFP-BOLD temporal correlations by assessing the noise level in our rsfMRI data. To this end, we evaluated the contrast-to-noise ratio (CNR) of our BOLD signal by leveraging the information that the spatial correlation of averaged M1 RSN maps (i.e. low-noise data) is 0.95 (Fig. 1K) while that of unaveraged RSN maps (i.e. high-noise data) is 0.58 (Fig. 3E). Based on this difference, we simulated two fixed signals with the true correlation of 0.95 (Fig. S6A). For each signal, random noise at a given CNR level was added and this process was repeated 159 times (i.e. equal to # of scans in our study). At each CNR level, the CC was calculated either based on the averaged signals from all 159 trials (i.e. low-noise data, Fig. S6A), or on signals of individual trials (i.e. high-noise data) before the resulting CCs were averaged across trials. To better mimic real BOLD/electrophysiology time series that are different across trials, for trial-wise correlations, two new signals with the same true correlation (i.e. 0.95) were simulated each trial. As expected, lower CNR gives lower apparent trial-wise CC values. Interestingly, we found that the trial-wise apparent CC of 0.58 with the true CC of 0.95 corresponds to the CNR at 1.3 (Fig. S6B-C), consistent with the CNR of BOLD contrast reported in the literature (Atkinson et al., 2008). At this CNR level, we estimated that the true BOLD-LFP temporal correlation in the M1 should be ~ 0.59 (R^2^ = 0.35, Fig. S6D-F) when the apparent correlation is 0.37 as measured by our data (Figs. 3A, 3E). Our simulation also indicates the difference in the number of data points (1200 in temporal correlation calculation vs 6157 in spatial correlation calculation) has a negligible influence on CC values (Fig. S6). This simulation shows that after removing the noise effect, the gamma-derived spatial map can explain up to 90% of variance in the BOLD-based seedmap, whereas the gamma power time information can only explain up to ~ 35% of variance in the BOLD time series.

All these data collectively indicate that the temporal information in the LFP signal has much lower predictive value to the local rsfMRI signal. These results are consistent with previous reports of relatively weak temporal correlations between gamma power and hemodynamic signals at rest obtained using different imaging modalities (Scholvinck et al., 2010; Winder et al., 2017).

### Regressing out LFP powers does not affect RSN spatial patterns

**Figure 4:**
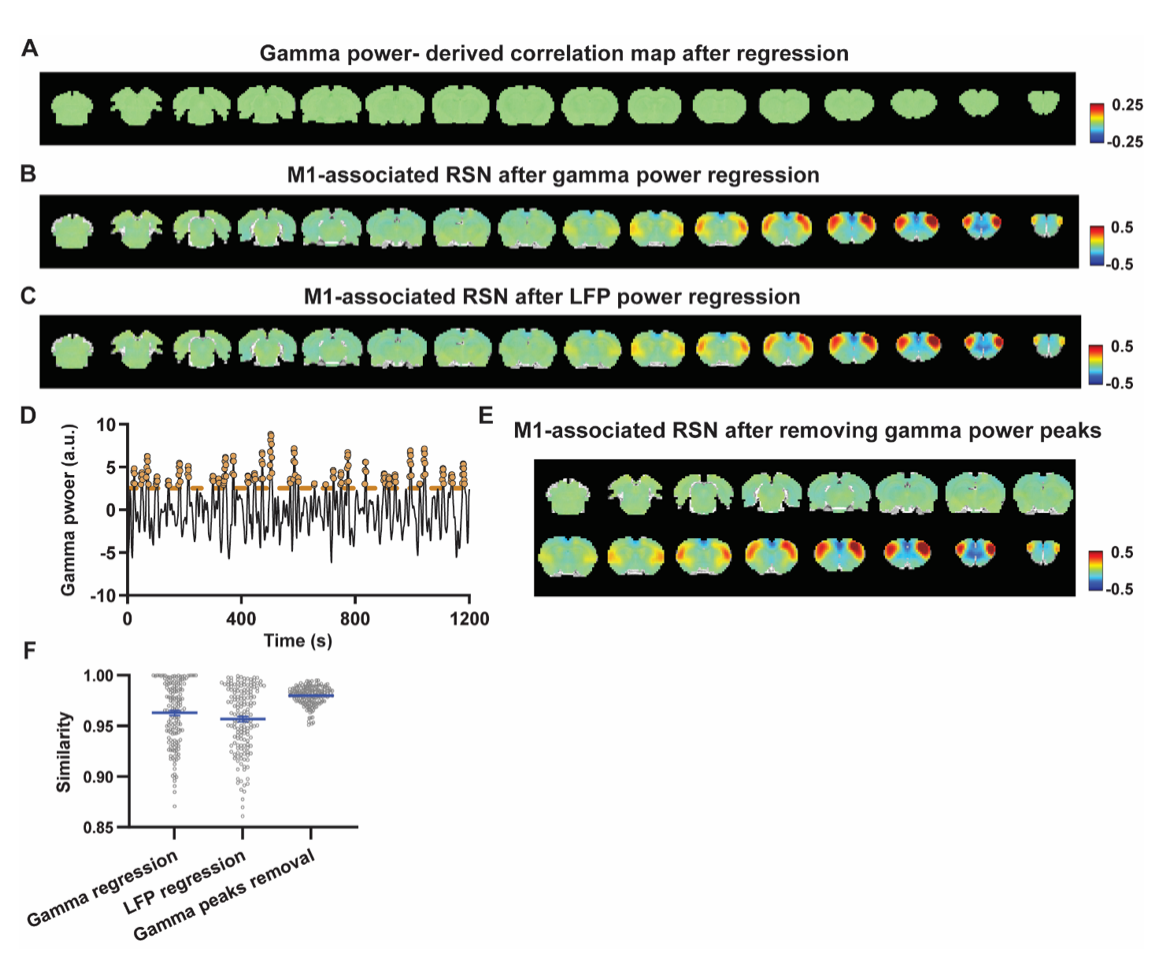
Impact of removing the electrophysiology signal on M1 RSN spatial patterns. **A)** Gamma power-derived correlation map after the HRF-convolved gamma power in the M1 is regressed out from rsfMRI signals of all brain voxels. **B)** M1 seedmap after the HRF-convolved gamma power is voxel-wise regressed out from rsfMRI signals. **C)** M1 seedmap after all five LFP band powers were voxel-wise regressed out from rsfMRI signals using soft regression. **D)** Peaks of HRF-convolved gamma power in one representative scan. **E)** M1 seedmap after 15% rsfMRI time points corresponding to gamma peaks were removed. **F)** Spatial similarity of M1 seedmaps before and after gamma power regression, regression of all LFP band powers or gamma peak removal.

**Figure 5:**
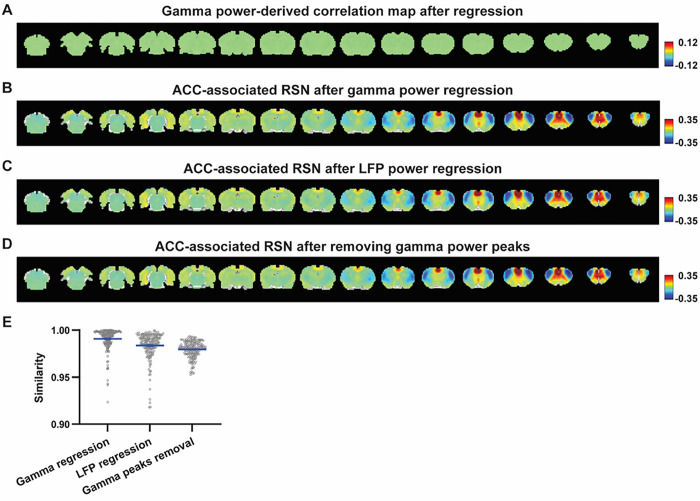
Impact of removing the electrophysiology signal on ACC RSN spatial patterns. **A)** Gamma power-derived correlation map after the HRF-convolved gamma power in the ACC is regressed out from rsfMRI signals of all brain voxels. **B)** ACC seedmap after the HRF-convolved gamma power is voxel-wise regressed out from rsfMRI signals. **C)** ACC seedmap after all five LFP band powers were voxel-wise regressed out from rsfMRI signals using soft regression. **D)** ACC seedmap after 15% rsfMRI time points corresponding to gamma peaks were removed. **E)** Spatial similarity of ACC seedmaps before and after gamma power regression, regression of all LFP band powers or gamma peak removal.

Given the lower predictive value of the LFP power on the local rsfMRI signal, we ask to what extent the temporal information of LFP powers affect the RSN spatial patterns. The M1 (or ACC) gamma-band power, after convolving with HRF, was regressed out from the rsfMRI signals of all brain voxels. As expected, the spatial patterns of gamma power-derived correlation maps observed in Figs. 1E & 2A disappeared after the regression (Figs. 4A, 5A). However, this regression process minimally altered the M1/ACC seedmap (Figs. 4B, 5B). This result remains the same when the powers of all LFP bands were voxel-wise regressed out from rsfMRI signals using soft regression (Figs. 4C, 5C).

To examine whether the regression process is disproportionally dominated by time points with the largest LFP amplitude (i.e. outliers), we recalculated the M1/ACC seedmaps after removing rsfMRI volumes corresponding to peaks in the M1/ACC gamma power (i.e. time points with the signal amplitude above 85 percentile in HRF-convolved gamma power of each scan, Figs. 4D, 4E & 5D). The spatial similarities between the seedmaps before and after M1/ACC gamma power regression, all band power regression, or peak removal are summarized in Figs. 4F & 5E, showing that the removal of gamma power has minimal impact on the M1/ACC RSN maps.

Considering that regression is a linear process, to control for the possible nonlinear relationship between band-specific LFP powers and the rsfMRI signal, we voxel-wise calculated the mutual information between band-limited LFP powers and rsfMRI signals for all brain voxels (Fig. S7). The data show limited mutual information between any band-specific power and voxel-wise rsfMRI signals, indicating that the nonlinear component between BOLD and electrophysiological signals does not play a major role in RSN spatial patterns. These data collectively indicate the temporal fluctuations of LFP have marginal effects on BOLD-derived RSN spatial patterns.

### Disparity in temporal and spatial correlations is consistent in different physiologic states

To determine whether the disparity between temporal and spatial correlations of resting-state LFP and fMRI signals we observed is a specific phenomenon under anesthesia or can be generalized to different physiologic states, we concurrently collected electrophysiology and rsfMRI data in awake rats. Despite the drastic change in the physiologic state, we again observed higher spatial correlations between LFP-derived maps (Fig. S8A) and the seedmap (Fig. S8B) in the M1, which gradually changed from negative correlations in low-frequency bands to positive correlations in high-frequency bands, revealed both in conventionally defined bands (Figs. S8C-G) and 1-Hz bands (Fig. S8H). This trend is generally maintained in the ACC, despite that the spatial correlations in low-frequency bands are diminished (Fig. S9). Similar to the results in Fig. 3, weaker but significant temporal correlations between the rsfMRI signal and HRF-convolved gamma band power were observed in unanesthetized rats (one sample t-tests; for M1, delta: CC = −0.05, p = 2.3 × 10^−4^; theta: CC = −0.06, p = 4.7 × 10^−6^; alpha: CC = −0.06, p = 4.6 × 10^−5^; beta: CC = −0.032, p = 3.0 × 10^−3^; gamma: CC = 0.04, p = 0.02; for ACC, delta: CC = 0.02, p = 0.29; theta: CC = 0.0009, p = 0.96; alpha: CC = −0.02, p = 0.31; beta: CC = 0.006, p = 0.66; gamma: CC = 0.10, p = 9.52 × 10^−7^; number of scans = 50). Scan-wise comparison between temporal and spatial correlations are shown in Fig. S10. Overall, we found that the magnitudes of both spatial and temporal correlations are generally lower in the awake state compared to the light sedation state, likely due to larger variances from motion and/or other physiological fluctuations, as well as a much lower number of scans (50 awake scans, 159 light-sedation scans for M1 and 172 light-sedation scans for ACC; number of scans = 159), but the pattern of lower temporal but higher spatial correlations between gamma power and the rsfMRI signal remain consistent.

Regressing out gamma-band power or powers of all LFP bands again did not significantly alter the seedmaps for both ACC and M1 in awake rats (Fig. S11). Taken together, these results show that all major findings in lightly anesthetized rats shown above can be replicated in awake rats, suggesting that these results are not specifically related to the effects of anesthesia.

### RSNs are driven by electrophysiology-invisible brain activities

**Figure 6:**
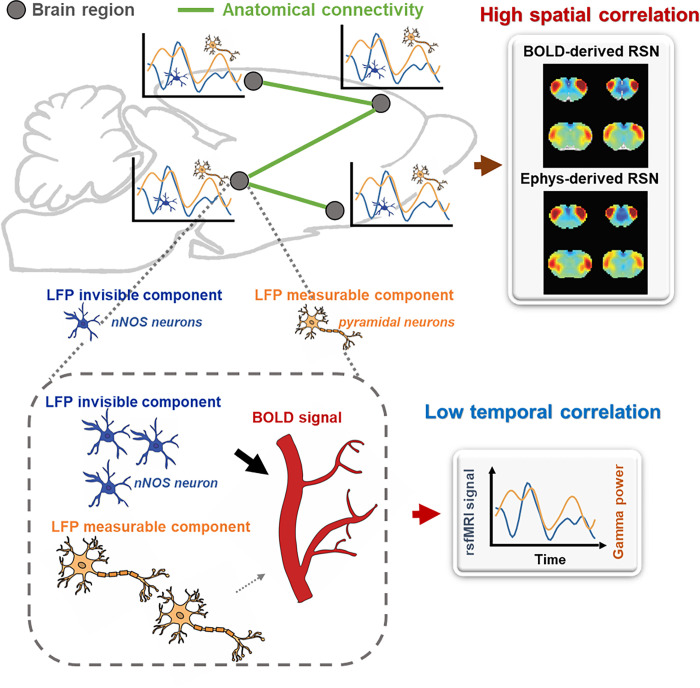
Proposed theoretic model that can explain the disparity in spatial and temporal correlations between resting-state electrophysiology and fMRI signals.

Our data reveal that the LFP signal can reliably derive RSN patterns that are highly consistent with BOLD-based seedmaps. On the other hand, we found that the temporal information of the LFP signal only minimally impacts the RSN spatial pattern. To reconcile this contradiction, here we propose a theoretical model that can possibly explain this apparent paradox, described as follows:

Brain activities include components that can be measured by electrophysiology and components that are electrophysiology invisible. Electrophysiology-invisible brain activities, such as activities of nNOS neurons and/or parvalbumin (PV) interneurons (Vo et al., 2023), are actively involved in NVC, and play a dominant role in the rsfMRI signal, whereas neural activity measured by electrophysiology has minimal effects on the rsfMRI signal. At the resting state, these two components are not necessarily synchronized, leading to low temporal correlations between electrophysiology and rsfMRI signals. Another factor that can contribute to low LFP-BOLD temporal correlations is neuromodulations from distal modulator nuclei (e.g. locus coerulues and/or basal forebrain), which have strong vasoactive effects but may not commensurately affect the electrophysiology activity. Meanwhile, signaling of electrophysiology activities as well as that of electrophysiology-invisible, and thus BOLD activities are both constrained by the same anatomical pathways, allowing the two signals to separately generate similar RSN spatial patterns which can reflect both direct and indirect anatomical connectivity. The model is summarized in Figs. 6 and S13. More details of the model will be discussed in the next section.

## Discussion

Although BOLD-based RSNs have been widely investigated in multiple species (Damoiseaux et al., 2006; Lu et al., 2012; Mantini et al., 2011) and applied in health and disease, the interpretation of interareal spontaneous BOLD synchronization is not fully comprehended. To gain more insight into this issue, here we systematically analyzed electrophysiology and rsfMRI signals simultaneously recorded in lightly anesthetized and awake animals. We found that both electrophysiology and rsfMRI signals can derive highly consistent brainwide RSN patterns, but the temporal information of the LFP signal only minimally contributes to the BOLD-based RSN spatial pattern. These paradoxical results, as well as those from literature studies can possibly be reconciled by the theoretical model we propose. These data and the model provide a completely new perspective for our interpretation of the neural basis underlying the resting-state BOLD signal.

The spatial correspondence between BOLD- and electrophysiology-derived RSNs has been repeatedly reported using multiple methodologies across different physiological states in both humans and animals. Studies utilizing electroencephalography (EEG) or electrocorticography (ECoG) in humans demonstrate that RSNs derived from the power of multiple-site electrophysiological signals show similar spatial patterns to classic BOLD-based RSNs such as the default-mode network (Hacker et al., 2017; Kucyi et al., 2018). In addition, simultaneous recordings of resting-state calcium and fMRI signals in awake rats show highly consistent spatial patterns between calcium- and BOLD-associated RSNs (Ma et al., 2022). Millisecond-timescale voltage-sensitive dye imaging reveals similar sensory-evoked and hemisphere-wide activity motifs represented in spontaneous activity in lightly anesthetized and awake adult mice (Mohajerani et al., 2013). All these data also well agree with the notion that the spatial patterns of RSNs are highly consistent with known functional systems and activation patterns observed in task-based studies (Biswal et al., 1995; Glasser et al., 2016; Hampson et al., 2002; Lowe et al., 1998; Smith et al., 2009), as well as patterns of structural networks (Andrews-Hanna et al., 2010; Greicius et al., 2009; Lowe et al., 2008). Taken together, previous studies and our data indicate consistent spatial patterns of RSNs can be derived from both electrophysiology and rsfMRI measurements, strongly suggesting that RSNs originate from neural activities.

Previous studies also demonstrate that RSNs are very likely constrained by axonal projects. Consistent sensory-evoked and hemisphere-wide activity motifs in mice revealed using voltage-sensitive dye imaging mentioned above are indeed defined by regional axonal projections (Mohajerani et al., 2013). Our previous work in awake rats also demonstrate high spatial consistency between RSNs and anatomical connectivity patterns in thalamocortical networks (Liang et al., 2013). In the present study, we compared the RSNs (i.e. seedmaps) of M1 and ACC to their anatomical networks defined by the axonal projection patterns obtained from the database of Allen Brain Institute (Fig. S12, (Oh et al., 2014)). Our data show that RSNs revealed by either the BOLD or LFP signal well resemble the corresponding anatomical networks. Taken together, these data indicate that RSNs are most likely constrained by the backbone of structural connectivity. It has to be pointed out that RSNs measured by functional connectivity can reflect both direct and indirect connectivity. Therefore, RSNs do not necessarily have completely identical spatial patterns as the corresponding anatomical networks (Honey et al., 2009).

In contrast to the seemingly strong LFP-BOLD relationship inferred from their high spatial correlations, we observed appreciably lower (but significant) temporal correlations between the two signals from the same recording sites (M1 and ACC). This result is corroborated by the finding that regressing out LFP power in any band does not significantly affect RSN spatial patterns, indicating that the contribution of temporal variations of the LFP signal to RSN spatial patterns is minor. Despite the apparent discrepancy, these findings are in fact supported by several literature studies. In a well-controlled experiment, Winder et al. demonstrated weak but significant correlations between cerebral blood volume (CBV) changes measured by intrinsic optical imaging and spontaneous gamma band LFP or multiunit activity in the barrel cortex in awake, head-fixed mice during rest (Winder et al., 2017). Importantly, they also observed persistent spontaneous fluctuations in CBV after blocking local neural spiking and glutamatergic input, as well as noradrenergic receptors, indicating that hemodynamic signal fluctuations may not necessarily reflect local ongoing electrophysiology activity (Winder et al., 2017). These data are in line with comparably low temporal correlations between gamma band power and the rsfMRI signal in monkeys (Scholvinck et al., 2010; Shmuel and Leopold, 2008). Data published in our group also show similarly low temporal correlations between spontaneous calcium peaks, a measure of neural spiking activity, and the rsfMRI signal in awake rats (Ma et al., 2022). All these data collectively suggest that although the temporal correlation between electrophysiology and rsfMRI signals is significant, the effect size of this correlation should be small. Notably, our results are different from two earlier studies in isoflurane-anesthetized rats, which found that the LFP power in the primary somatosensory cortex was highly correlated with the BOLD fluctuation (Liu et al., 2011; Pan et al., 2011). This discrepancy might be attributed to the effect of brain-wide synchronization during burst suppression in deeply anesthetized states.

Why would electrophysiology and rsfMRI signals exhibit unmatched spatial and temporal correlations? Our model hypothesizes that the rsfMRI signal is mainly driven by electrophysiology-invisible brain activities that are actively involved in NVC. Indeed, LFP is a composite signal that records various neural activities, such as synaptic potentials and voltage-gated membrane fluctuations, reflecting the input and local neural processing of a particular brain region. However, electrophysiology cannot measure activities from certain cell populations, while these electrophysiology-invisible components can trigger vasoactive responses and significantly contribute to the rsfMRI signal. For instance, the electrical activity of nNOS neurons is not detectable by electrophysiology measurement because the nNOS neuron population is very small, but it strongly contributes to NVC. Chemogenetic or pharmacological stimulation of nNOS neurons causes vasodilation without detectable changes in LFP (Echagarruga et al., 2020). In addition, stimulation of PV neurons leads to release of substance P, which can activate a subset of nNOS neurons and result in vasodilation without electrophysiological changes (Endo et al., 2016; Vo et al., 2023). These studies collectively suggest some electrophysiology-invisible activities can significantly drive the rsfMRI signal. Exclusively identifying all electrophysiology-invisible sources that contribute to the rsfMRI signal is beyond the scope of this work. However, a key point is that as the resting state LFP and electrophysiology-invisible (and thus rsfMRI) signals reflect different components of brain activities, they can be minimally synchronized and display low temporal correlations. Another possible factor that can contribute to low BOLD-LFP temporal correlations is direct modulation of the vasculature from distant modulator nuclei (e.g. locus coerulues and/or basal forebrain). Neuromodulators, such as norepinephrine (NE) (Bekar et al., 2012; Kim et al., 2016) and acetylcoline (Ach) (Sato and Sato, 1995), have vasoconstrictor/vasodilatory effects that are spatially targeted in distributed brain regions, and thus can modulate the brain-wide rsfMRI signal. However, these neural modulation effects may not be commensurately reflected from the electrophysiology signal, leading to low BOLD-LFP temporal correlations (see a schematic diagram in Fig. S13). On the other hand, as the signaling of LFP- and electrophysiology-invisible components in functional networks are constrained by the same anatomical connectivity structure (Liu et al., 2018a; Liu et al., 2018b), the LFP- and BOLD-derived RSN spatial patterns can be highly similar and thus have high spatial correlations.

Our model can potentially also explain high spatial and temporal correlations when brain activation is evoked by external stimulation (Winder et al., 2017). At the evoked state, both LFP (and spiking activity) and electrophysiology-invisible components are temporally modulated by the same external stimulation paradigm, leading to both high temporal and high spatial correlations between fMRI and electrophysiology signals.

## Summary

Our study revealed that BOLD-based RSNs can be reliably derived by the electrophysiology signal, but they might be dominantly attributed to the electrophysiology-invisible signal. This study provides a new interpretation of RSNs. However, this new concept of RSN signaling does not in any way argue against the importance of BOLD-based RSNs and the rsfMRI method. In fact, it makes fMRI even more important than previously thought because it might provide a new signal that traditional electrophysiology measures cannot provide.

## Methods and Materials

### Animals

All experiments in the present study were approved by and conducted in accordance with guidelines from the Pennsylvania State University Institutional Animal Care and Use Committee (IACUC). Adult male Long-Evans rats weighing 300–500g were obtained from Charles River Laboratory (Wilmington, MA). Animals were housed in Plexiglas cages with food and water given ad libitum. The ambient temperature was maintained at 22–24°C under a 12h light :12h dark cycle.

### Surgery

MR-compatible electrodes were implanted in animals with aseptic stereotaxic surgeries. The rat was briefly anesthetized with isoflurane before receiving intramuscular injections of ketamine (40 mg/kg) and xylazine (12 mg/kg). Baytril (2.5mg/kg) and long-acting buprenorphine (1.0mg/kg) were administered subcutaneously as antibiotics and analgesics, respectively. The animal was then endotracheal incubated and ventilated with oxygen using the PhysioSuite system (Kent Scientific Corporation). Body temperature was monitored and maintained at 37°C with a warming pad placed underneath the animal (PhysioSuite, Kent Scientific Corporaition). Heart rate and SpO_2_ were continuously monitored using a pulse oximetry (MouseSTAT^®^ Jr, Kent Scientific Corporation) throughout the surgery. After performing craniotomies over the right ACC (coordinates: anterior/posterior + 1.5, medial/lateral + 0.5, dorsal/ventral − 2.8) and the left M1 (coordinates: anterior/posterior + 3.2, medial/lateral + 3, dorsal/ventral − 2.8), two MR-compatible electrodes (MRCM16LP, NeuroNexus Inc) were carefully implanted into the ACC and M1, respectively. The reference and grounding wires from each electrode were wired together and connected to one of the two silver wires placed in the cerebellum. At last, the skull was sealed with dental cement. After surgery, the animal was returned to the homecage and allowed to recover for at least one week before any experiment.

### Acclimation for awake imaging

Rats were restrained using a custom-designed restrainer during awake imaging sessions. To minimize stress and motion during the imaging process, animals underwent a 7-day acclimation procedure to the restrainer as well as the MRI environment and scanning noise. The duration of the acclimation procedure was gradually increased from 15 min on the first day to 60 min on days 4–7 days (i.e., 15 min on day 1, 30 min on day 2, 45 min on day 3, and 60 min on days 4–7). More details of the acclimation procedure can be found in previous publications from our laboratory (Liang et al., 2012) and other research groups (Bergmann et al., 2016; Chang et al., 2016).

### Simultaneous rsfMRI and electrophysiology recordings

All rsfMRI experiments were conducted on a 7T Bruker 70/30 BioSpec system running ParaVision 6.0.1(Bruker, Billerica, MA) using a homemade single loop surface coil at the *high field MRI facility* at the Pennsylvania State University. During each fMRI session, T2*-weighted rsfMRI images covering the entire brain were obtained using an echo planar imaging sequence with the following parameters: repetition time (TR) = 1 s; echo time (TE) = 15 ms; field of view = 3.2 × 3.2 cm^2^; matrix size = 64 × 64; slice number = 20; slice thickness = 1mm; volume number = 1200. Five to ten scans were repeated within each session. T2-weighted anatomical images were also acquired using a rapid acquisition with relaxation enhancement (RARE) sequence with the following parameters: TR = 3000 ms; TE = 40 ms; field of view = 3.2 × 3.2 cm^2^; matrix size = 256 × 256; slice number = 20; slice thickness = 1 mm; repetition number = 6.

Six rats with two electrodes implanted in the ACC and M1 were imaged in both awake and lightly sedated states in separate fMRI sessions. Two additional rats with an electrode only implanted in the ACC were imaged in the light sedation state. For both states, animals were restrained throughout the imaging session. In the light sedation state, the animal was sedated with the combination of low-dose dexmedetomidine (initial bolus of 0.05 mg/kg followed by a constant infusion at the rate of 0.1 mg·kg^−1^·h^−1^) and low-dose isoflurane (0.3%). Artificial tears were applied to protect the animal’s eyes from drying out. Body temperature was maintained at 37°C using warm air and was monitored using a rectal thermometer.

Before imaging, the implanted electrodes were connected to MR-compatible LP16CH headstages and a PZ5 neurodigitizer amplifier (Tucker Davis Technologies (TDT) Inc, Alachua, FL). Electrophysiology recording began 10 min before rsfMRI data acquisition and continued until the end of the imaging session using a TDT recording system and an RZ2 BioAmp Processor (TDT Inc, Alachua, FL). The raw, unfiltered electrophysiology signal was sampled at 24414 Hz and stored using the TDT Synapse software on a WS8 workstation.

### rsfMRI and electrophysiology data preprocessing

All data preprocessing and analysis were performed using MATLAB (Mathworks, Natick, MA). First, the movement of each rsfMRI volume was estimated using the framewise displacement (FD). For the awake imaging data, volumes with FD > 0.1 mm and their adjacent preceding and following volumes were removed. If > 25% of volumes in a scan were scrubbed, the entire scan was excluded from further analysis. For rsfMRI data collected in the lightly sedated state, scans with any volume that had FD > 0.1 mm were removed from further analysis. Subsequently, data were preprocessed using the following steps: co-registration to a defined atlas, motion correction (SPM12), spatial smoothing using a Gaussian kernel (FWHM = 0.75 mm), voxelwise nuisance regression with the regressors of motion parameters as well as signals from the white matter and ventricles, and the global brain signal, and, lastly, bandpass filtering (0.01–0.1 Hz).

Raw electrophysiology data were preprocessed to remove the MR interference using a template regression method as previously described (Tu and Zhang, 2022). Briefly, the raw electrophysiology signal for each scan was first aligned to the corresponding rsfMRI scan, and segmented for each imaging slice based on the starting time of the scan. Next, an MRI interference template for each rsfMRI slice acquisition was obtained by averaging the electrophysiology data across all slices from all rsfMRI volumes. The template was further aligned to the electrophysiology data for each slice acquisition using cross correlation and was then linearly regressed out from the raw electrophysiology data. In addition, a series of notch filters for harmonics of the power supply (60 Hz and multiples of 60 Hz) and slice acquisition (20 Hz and multiples of 20 Hz) were applied to further denoise the data.

### Data analysis

The LFP power was obtained by bandpass filtering preprocessed electrophysiology data in the frequency range of 0.1–300 Hz. Based on the conventional LFP band definition (delta: 1–4 Hz, theta: 4–7 Hz, alpha: 7–13 Hz, beta: 13–30 Hz, gamma: 40–100 Hz) (Lu et al., 2016; Zhang et al., 2020), the LFP band power was computed using the MATLAB function *spectrogram* with a window size of 1 s and a step size of 0.1 s. To investigate the relationship between the LFP and rsfMRI signals, the time course of band-specific LFP power was convolved with a hemodynamic response function (HRF, p = [4 4 1 1 6 0 32] for function *spm_hrf*) to generate the corresponding LFP-predicted BOLD signal. The HRF used was specific to rodents with a shorter onset time and time-to-peak as a faster HRF was reported in rats relative to humans (Tong et al., 2019). The temporal relationship between the LFP and fMRI signal was quantified using the Pearson correlation between the HRF-convolved LFP band power and the regionally averaged rsfMRI time course from voxels surrounding the implanted electrode for each site.

The LFP-derived spatial correlation maps for the M1 and ACC were respectively generated by computing voxel-wise Pearson correlations between each HRF-convolved LFP band power and brain-wide rsfMRI signals. The seedmaps for the M1 and ACC were respectively obtained by calculating the voxel-wise Pearson correlations between the regionally averaged rsfMRI time course for each seed and the rsfMRI signals of all brain voxels.

To determine the contribution of LFP power to BOLD-based RSFC, we removed the LFP powers from voxel-wise fMRI signals and then recalculated RSNs. Given that the gamma signal might be the most related to the rsfMRI signal, the time course of gamma-band power (convolved with HRF) was linearly regressed out from rsfMRI signals of all brain voxels. To examine the potential contributions of other LFP bands, all five LFP band powers (each convolved with HRF) were “softly” removed from voxel-wise rsfMRI signals, meaning only the unique components in five bands were regressed out but the shared components were maintained. Specific details of soft regression can be found in (Griffanti et al., 2014). This method can avoid ‘over regression’ when multiple regressors are involved particularly when regressors are correlated between themselves. Lastly, we removed rsfMRI volumes corresponding to peaks in the M1/ACC gamma power. The seedmaps of M1 and ACC were recalculated and compared before and after removing the LFP signal.

### Simulation

We simulated two fixed signals with the true Pearson correlation of 0.95. The first signal was generated using MATLAB function rand with 10000 data points. The second signal with a defined correlation with the first signal (i.e. 0.95) was obtained based on the equation below:

$$B=A^{*}\text{Corr}\;+\sqrt{1-{Corr}^{2}}*N(0,1)$$

in which A represents the first signal, Corr is the desired Pearson correlation coefficient, and N(0,1) represents random values with the mean equal to 0 and standard deviation equal to 1.

For each signal, random noise was added to achieve a contrast-to-noise ratio (CNR) ranging from 0.1 to 5 with the step size of 0.1. CNR was quantified by the standard deviation of the signal over the standard deviation of the noise. This process was repeated for 159 times (equal to the # of scans in the present study). The noise-added signals were resampled to either 1200 or 6157 data points, which corresponded to the total number of time points used to calculate temporal correlations and total number of brain voxels used to calculate spatial correlations, respectively, in our study. Pearson correlations between the resampled signals either based on the averaged signals from all 159 trials or on individual trials were calculated.
